# Efficiency of primary care in rural Burkina Faso. A two-stage DEA analysis

**DOI:** 10.1186/2191-1991-1-5

**Published:** 2011-07-20

**Authors:** Paul Marschall, Steffen Flessa

**Affiliations:** 1 University of Greifswald, Faculty of Law and Economics, Department of Health Care Management, Friedrich-Loeffler-Str. 70, D-17489 Greifswald, Germany

**Keywords:** Burkina Faso, DEA, Efficiency, Nouna, Primary Care

## Abstract

**Background:**

Providing health care services in Africa is hampered by severe scarcity of personnel, medical supplies and financial funds. Consequently, managers of health care institutions are called to measure and improve the efficiency of their facilities in order to provide the best possible services with their resources. However, very little is known about the efficiency of health care facilities in Africa and instruments of performance measurement are hardly applied in this context.

**Objective:**

This study determines the relative efficiency of primary care facilities in Nouna, a rural health district in Burkina Faso. Furthermore, it analyses the factors influencing the efficiency of these institutions.

**Methodology:**

We apply a two-stage Data Envelopment Analysis (DEA) based on data from a comprehensive provider and household information system. In the first stage, the relative efficiency of each institution is calculated by a traditional DEA model. In the second stage, we identify the reasons for being inefficient by regression technique.

**Results:**

The DEA projections suggest that inefficiency is mainly a result of poor utilization of health care facilities as they were either too big or the demand was too low. Regression results showed that distance is an important factor influencing the efficiency of a health care institution

**Conclusions:**

Compared to the findings of existing one-stage DEA analyses of health facilities in Africa, the share of relatively efficient units is slightly higher. The difference might be explained by a rather homogenous structure of the primary care facilities in the Burkina Faso sample. The study also indicates that improving the accessibility of primary care facilities will have a major impact on the efficiency of these institutions. Thus, health decision-makers are called to overcome the demand-side barriers in accessing health care.

## Background

Improving the health status of people in low-income countries is a very important human goal which has been on the global agenda for a long time [1]. However, for many years the transfer of financial funds was regarded as a sufficient strategy to safeguard that health services provide effective health care [2,3]. The efficient utilization of these funds was strongly neglected by international and local decision-makers as well as by scientists [4,5], but meanwhile there is agreement that low-income countries can only achieve the health-related Millennium Development Goals (MDGs) if research and health-policy focus on efficiency [6,7].

In industrialised countries, measuring efficiency, benchmarking and consequent improvement of performance have become a standard and a wide variety of instruments was developed, ranging from simple ratios and unit costing to more complex methodologies, such as Data Envelopment Analysis (DEA) and Stochastic Frontier Analysis [8]. In particular, DEA has proven to be of great practical value for the calculation of the relative efficiency, the benchmarking process [9] and the performance enhancement of different organisations [10], such as energy enterprises [11], universities [12], and health care facilities [13]. In the industrialised world, DEA is frequently utilized as a benchmarking tool for health care institutions [14] and helps to determine the strengths and weaknesses of facilities in order to improve the ratio of inputs and outputs.

In spite of the basic necessity to avoid waste of scarce resources in health care institutions, health economic research in Africa is focussed on specific interventions programmes or the entire health care system [15,16]. Benchmarking of service providers and in particular Data Envelopment Analysis are very rarely performed in Africa. Most of the published studies concentrate on hospitals of the same level, for instance in Angola [17], Botswana [18], Ghana [19], and Namibia [20], or large health centres operating almost at the hospital level, such as in Ghana [21], Sierra Leone [22], and Kenya [23]. Very little is known about the efficiency of small primary care facilities in African countries, although these institutions treat the majority of patients.

In this paper we present a DEA analysis of small primary care facilities in the health district of Nouna, Burkina Faso. These institutions are the prime source of medical services for the population of this health district, and every contribution to measure and increase the efficiency of these facilities will have a direct impact on human life. The study is based on previous research on the efficiency of primary care facilities in Nouna health district [24]. We expand the efficiency measurement by a comprehensive second stage, in which the efficiency scores estimated at the first stage are explained in a regression with environmental variables as independent variables. We are motivated to perform this analysis as we expect to determine the reasons for poor efficiency of some primary care facilities and to find parameters to improve the performance of these institutions. Another intention of this paper is the proof that advanced efficiency analysis is a feasible and meaningful tool of health systems research in the African context.

The structure of this paper is as follows. In the next section, the methodological issues are presented. We provide some background information on the study area and outline the adopted two-stage DEA analysis. This is followed by the presentation of our results. Subsequently, we hold a discussion on the corresponding implications. The final section contains our concluding remarks.

## Methods

### Study Site

The study was carried out in the Nouna Health District, which is identical to the administrative province of Kossi in Northwestern Burkina Faso, a land-locked country in Western Africa with an estimated population of about 14.7 million, 74% of them live in rural areas. Kossi covers an area of 7,464 square kilometres [25]. This predominantly rural, semi-arid area at the edge of the Sahel zone has a population of almost 279,000 inhabitants. Most of the people there are illiterate, extremely poor and work as subsistent farmers, producing crops like millet and sorghum. The most important ethnic groups are Dafing/Marka, Bwaba, Mossi and Peulh [26]. The people in this region are predominantly Muslim (65%), with some percentage of the population being Christian (29%), and 6% adherer to traditional beliefs respectively.

Formal health services are provided by 24 primary care facilities (*Centre de Santé et de Promotion Sociale*, CSPS), one dispensary and a small hospital (*Centre Medical avec Antenne Chirurgicale*, CMA) in Nouna town. The primary care facilities (CSPS) provide first-line services at the village level and are primarily located in rural, peripheral areas of Burkina Faso [27]. They are usually staffed with a team of two to four health workers and one to three unqualified volunteers. They are responsible for a catchment area of 5 to 23 villages. Each CSPS provides basic outpatient services (incl. a maternity unit) and is linked to a public pharmacy. There are also vaccination programmes which are operated locally in the villages. The CMA, which is equipped with nearly 100 beds with surgical facilities, is the first referral level for the primary care facilities [6].

### Basic DEA Model

Data Envelopment Analysis is rooted in the concept of efficiency. In general, this term reflects the degree of success with which an organisation uses its inputs/resources *x *to produce outputs *y *of a given quality. This can be assessed in different categories of efficiency. *Technical *efficiency is determined by comparing the difference between the observed ratio of combined quantities of an organization's output to input and the ratio achieved by best practice. Producing the maximum output or consuming the minimum inputs, as compared to what is technically feasible, is an essential step for service providers to be able to meet their objectives best. According to this concept an organisation or facility (decision-making unit, DMU) is efficient, if it operates on its corresponding production possibilities frontier. Inefficient producers operate below it. Generally, the best practice frontier can be determined either by using parametric approaches, which are based on regression analysis, or by applying nonparametric techniques. Data Envelopment Analysis, which was proposed by Charnes et al. [28] in its present form, is linked with the concept of *relative *efficiency of similar entities. According to Banker and Maindiretta [29] it offers certain important advantages over parametric methods: First, DEA does not impose the assumption of any functional form on the relationship between inputs and outputs. This attribute is especially useful for cases in which the correspondence is not known or specified by theory. DEA uses linear programming to construct a piecewise efficiency frontier and must only be based on the minimum assumptions of monotonicity and convexity of the efficiency frontier. Second, DEA can be used not only to identify inefficient units, but also to estimate the degree of inefficiency. Third, it is possible to include multiple inputs and outputs in DEA, which is especially important for the analysis of health care services, whereas the weights are calculated within the DEA procedure. The DEA method allows variable and fixed inputs, whereas the variable inputs might change in the short run while the values of the fixed inputs are only allowed to be changed in the long run. The DEA approach allows each DMU to choose the optimum weightings for the outputs and inputs. Each DMU is considered in turn and its most favourable weights are selected. Special features provide the analysis' adjustment to the concrete problem setting.

However, the strengths of DEA lay the foundation of its weaknesses as well. As DEA is an empirically based estimation technique, it is sensible to outliers, error measurements and random influences in the data. DEA deems any deviation from the efficiency frontier to be the result of inefficiency. From the endogenous weighting system follows a second shortcoming. If the number of factors considered in the efficiency analysis is relatively high, the DEA approach may lead to substantial overestimates of the efficiency of DMUs. Nevertheless, DEA is probably because of its advantages the most appropriate technique currently available for measuring relative efficiency in health services [30]. The number of studies has increased dramatically over the past few years [31].

According to DEA the efficiency of a multiple-output, multiple-input DMU *k*, with *k *= 1, ..., *n*, can be presented as follows:(1)

whereas *u *measures the weight of each output *y_j_*(*j *= 1, ..., *s*), and *v *indicates the weight of each input *x_i_*(*i *= 1, ..., *m*) The efficient frontier of the group (*θ *= 1) is constituted by the most efficient DMUs to which the efficiencies of the remaining DMUs are related to. Best performers do not waste any input and can therefore be regarded as "peers" for entities with a weak evaluation and a less efficiency. This implies that the efficiency score *θ *falls between 0 and 1.

To estimate the efficiency frontier, different important options have to be fixed. The orientation of the models reflects the appropriate direction of optimisation. This reflects which kind of quantities managers have better under control. In some branches, the organisations may be given a fixed quantity of resources and asked to produce as much output as possible. In this case the output-oriented approach might be appropriate. By contrast, an input-oriented approach should be used, if a fixed level of output has to be reached by using a minimal quantity of inputs. Because DMUs might differ according to their size and the quantities of inputs used and outputs produced respectively, assumptions concerning the returns to scale have to be formulated. The Charnes, Cooper, Rhodes (CCR) model incorporates constant returns to scale in production. The efficiency measure (1) and the usual side conditions can be adjusted accordingly. To obtain a linear programming problem, the Charnes-Cooper transformation [32] can be used. The output-oriented linear programming envelopment for the DMU under evaluation *k *is:(2)

whereas *η *is defined based on the efficiency score of DMU k:(3)

and the vector *φ *represents intensity variables which indicate the necessary combination of efficient entities (reference unit or peer) for every inefficient DMU in order to form a "virtual unit" or benchmark that is on the frontier.

Based on the CCR approach several other models were developed which build a profound basis for efficiency analysis with different returns to scale, different envelopment surfaces and different ways to project inefficient entities to the efficient frontier. Banker et al. [33] formulated the Banker, Charnes, Cooper (BCC) model which evaluates solutions for non-increasing returns to scale, non-decreasing returns to scale or variable returns to scale. Whereas the CCR model only measures overall technical efficiency , the BCC model exclusively evaluates pure technical efficiency , because scale effects are taken into account. The comparison of CCR and BCC results enables to identify inefficiency which can be mitigated by increasing or decreasing the production volume resulting in a removal of scale inefficiencies. Thus, the ratio of the CCR and BCC efficiency measures will yield an estimate of the pure scale efficiency (SE) of DMU *k *[33], i.e.,(4)

indicating whether e.g. the different primary care facilities were operating on an efficient scale in producing their services. The optimal size of a DMU is reached when a marginal increase of all inputs (scale) leads to the same relative increase of outputs. The bigger the difference between the scale efficiency score of a DMU and full scale efficiency (*SE_k _*= 1), the more unfavourable are the consequences of scale. It tells us how much output of a DMU can be expanded until it is as efficient as the reference unit.

### Two-Stage DEA Analysis

There are many important DEA extensions [34]. Amongst others, one-stage DEA, which only involve standard DEA analysis, has been broadened by a second stage, in which regression technique is used to explain the efficiency scores, which were measured at the first stage. The classical linear regression model takes the form(5)

where *Y*, a dependent variable, is explained by a vector of independent variables *X_i_*. The *β_i _*are unknown regression coefficients, *β*_0 _represents a constant and *ε *is the error term reflected in the residuals. Regression equation (5) can be estimated by Ordinary Least Squares (OLS), which minimises the sum of squared distances between the observed responses in a set of data, and the fitted responses from the regression model. OLS yields best linear unbiased estimates (BLUE) on the assumption of independent identically distributed (iid) observations with constant mean and variance, if several important assumptions about the way in which the observations are generated are not violated [35]. If e.g. the relationship between dependent and independent variables is not linear or the dependent variable is limited in some way, OLS estimates are biased, even asymptotically. Based on diagnostic analyses or misspecification tests hints for a more adequate formulations can be detected.

Two-stage DEA aims at explaining the efficiency score *θ *by a set of environmental influences and other non-discretionary (sometimes causal) factors which are beyond the control of the facility managers. In order to get unbiased results it is of great importance to choose the most appropriate estimation technique. Especially the characteristics of the dependent variable *θ *have to be carefully studied.

Several authors have interpreted it *θ *as censored, limited to the interval ]0;1], thus using the (censored) Tobit model to analyse the relationship with explanatory variables [36]. Equation (5) has to be adjusted accordingly.

The Tobit model can thus be defined for DMU *k*:(6)

where  is an unobserved latent variable and *θ_k _*is the DEA score. *X_k _*is a (row) vector of observation-specific variables for DMU *k *that affect its efficiency score through the vector of parameters *β *to be estimated.

There is an ongoing discussion whether the Tobit model is really the appropriate functional form for performing the second stage analysis. Wheras Hoff [36] states that in most cases the Tobit approach will be sufficient in representing the second stage DEA models, McDonald [37] argues, that this approach might be inappropriate because the efficiency scores are not generated by a censoring process but are fractional data. Based on the results of post-estimation regression analyses it has to be decided which approach is more appropriate. In a recent paper Simar and Wilson [38] emphasised that these conventional approaches have severe shortcomings because the efficiency scores generated at the first stage are strongly dependent on each other in the statistical sense, and using them in a second stage regression might violate the basic model assumption required by regression. Because measurement errors which affect observations on the efficiency frontier can create complicated patterns of serial correlation for observations that lie within that portion of the frontier, they proposed a truncated regression approach with a bootstrap procedure instead. The latter is a computer-based method that is based on the idea of re-sampling from an original data to assign statistical properties for the efficiency scores [39]. We follow the estimation algorithm of Simar and Wilson [38] as there is currently no methodical alternative [40].

### Data

We used data collected by a comprehensive long-term cost information system which covers both supply and demand side, and which contains information about direct and indirect costs. The provider information system was established in 2003 and gathers costing data from every health facility within the district [41]. Since the year 2000, a household survey is operative in that area under demographic surveillance by the *Centre de Recherche en Santé de Nouna *(CRSN). The survey, known as Nouna Health District Household Survey (NHDHS), records information on a statistically representative sample of the population within the study area of the CRSN, which covers a considerably part of the Nouna health district, at regular intervals of time [42]. Both complementary information modules provide data that permit the calculating of the total tangible costs of illness in the complete health district.

At the first stage of the analysis we used data from all 25 CSPS which were operative in 2005 in the Nouna Health District. Three of them started their activity in the middle of that year. With the exception of immunisation, all other services which are regularly provided at the level of primary care facilities were offered. The activity data were adjusted accordingly. We excluded data from the pharmacies because Burkina Faso joined the Bamako Initiative in 1992 and they operate independently from the primary care facilities they are linked to.

In order to measure the CSPS efficiencies we restricted our analysis to four inputs and four outputs. As inputs we chose 1) Personnel costs in 2005 [US$], 2) CSPS building area [m^2^], 3) Depreciation of CSPS equipment in 2005 [US$] and 4) Vaccination costs in 2005 [US$]. Personnel costs were used as a proxy for the production factor labour. The structure of the health care personnel across the primary care facilities is quite similar concerning their qualification. In employment contracts for the public sector in Burkina Faso occupational skill and not working time determines individual earnings. Thus, personnel costs incorporate the qualification level. Most studies which assess efficiency in hospitals or bigger health facilities in developing countries use "number of beds" as an indicator for capital inputs. As hospitalisation in the CSPS occurs only in cases of emergency and for a very small length of stay (Ø < 1 day), beds are no appropriate indicator. Therefore the building area of the CSPS buildings and the annual depreciation costs for equipment in the primary care facilities were used. The lifetime of equipment was estimated using information from the ministry of health. Equipment with a value below 10,000 FCFA (18.60 US$) was depreciated at once. Vaccinating represents one of the most expensive activities which were performed at the level of primary health care.

We used four output measures which reflect the main activities performed in the primary care facilities: 1) general consultation and nursing care, 2) deliveries, 3) immunisation, and 4) special services, e.g. family planning, prenatal and postnatal consultations. All of these indicators are based on the number of cases thus they only reflect intermediate outputs.

Because DEA is very sensitive with regard to missing data and outliers, we pursued two strategies to adjust for the missing vaccination services at three CSPS: (1) Vaccination input and output data were balanced according to the catchment area. (2) Efficiency measurement was limited to three inputs and three outputs, excluding vaccination. Therefore, the combination of the measured indicators ensures in both cases adherence to the DEA convention that the minimum number of DMU observations should be greater than three times the number of inputs plus outputs [43].

Since the choice of a particular DEA model has effects on the study, the special situation in Nouna Health District has to be taken into account. There are good reasons for choosing "input-orientation" in industrialised countries because low demand for health services is not regarded as problem per se [44]. However, an "output-oriented" approach is appropriate for Nouna health district. Local committees (Comité de Gestion, COGES) which are responsible for the administration of the primary care facilities in Nouna Health District have only limited control over the volume of inputs. Decision-makers at the level of the health district and at the ministry of health take the relevant input decisions. They decide about the installation of a further CSPS, provide primal endowment and pay the staffs' salaries. In theory, COGES can use local revenue from user fees to finance additional staff or new equipment, but in reality these resources are insufficient.

To estimate the impact of non-discretionary factors on the efficiency of the primary care institutions we used 2005 data from the Nouna Health District Household Survey. Information about 7,345 individuals, who lived in the catchment area of eight CSPS, was available. The information about the name of the nearest CSPS was replaced by the corresponding DEA score. The exogenous variable selection was based on our hypothesis that problems on the demand side might influence the performance of the CSPS. According to a standard approach in health economics individuals produce their own health [45]. Their own preferences, which might be influenced by their social and cultural environment and a set of financial and other constraints, are responsible for their own health care seeking. On an aggregated level these behavioural patterns are accountable for the actual demand at the level of health facilities, which in turn might have consequences for the efficiency of the CSPS. These factors can be interpreted as barriers to health-care seeking. Therefore, we included variables which can be linked to that approach. Based on information about individual ownership of animals and local prices we created a livestock variable. Domestic animals represent accumulated savings. Additionally we collected information on durable goods (bicycles, radios, TV sets etc.) and combined them with local price information.

In addition, cultural background and religious beliefs might also influence individual health seeking behaviour. For instance, Marschall and Fleßa [24] reported that a specific conservative religious group (Islamic Wahabi movement) obviated some parts of modern health care. Particular Muslim factions shaped some communities' relation to immunisation questions. In their study about specific factors associated with the vaccination status in Nouna Health District, Sanou et al. [46] found out that children of Muslim families (controlling for economic status) have significantly lower rates of complete immunisation coverage in rural areas. Non-Muslims had almost twice the probability of being in the completely vaccinated group.

Finally, it can be assumed that ethnic affiliation can be important for not seeking modern healthcare. This information is also given in the Nouna Health District Household Survey.

## Results

### Descriptive results

Table 1 shows the results of the cost-of-illness information system of Nouna health district. It is obvious that the statistics have a wide range. According to the official standards of Burkina Faso a CSPS has to provide services for about 10,000 people within a radius of the catchment area of 10 kilometres [47-49], and it is in principal built and staffed according to a national standard for primary care facilities. However, in reality most of the peripheral entities are understaffed and have low annual personnel costs. In contrast, the CSPS in Djibasso and the dispensary in Nouna town have costs above 19,000 US$. The Djibasso CSPS is a previous *Centre medical *(CM), which was downgraded in its status to a primary care facilitiy, so that it has still the overheads of a CM although functionally working as a CSPS.

**Table 1:** Descriptive statistics of the input and output measures

	Mean	Standard Deviation	Minimum	Maximum
**Inputs:**				

Personnel Cost [US$]	6,282	4,956	3,124	24,768

Area [m^2^]	155	70	52	363

Equipment Depreciation [US$]	2,509	1,291	858	5,278

Vaccine [US$]	14,814	10,002	2,809	52,171

**Outputs:**				

Number of General Consultations	2,226	1,098	825	4,654

Number of Deliveries	369	226	40	897

Number of other Care	2,941	1,804	380	6,422

Number of Vaccinations	6,943	8,400	588	41,231

Also the floor area shows a wide range between 52 (Lekuy) and 363 (Nouna town) square metres. Differences in equipment costs (858 US$ - 2,509 US$) are mainly the consequence of the corresponding age of the institution, as expensive inventory is normally transferred at the date of opening the facility. Replacement of written-off equipment is the exception rather than the rule.

Most of the primary care facilities reported vaccine costs between 10,000 US$ and 20,000 US$. However the vaccination costs of CSPS Djibasso were nearly seven times higher than those in the catchment area of CSPS Kononkoira.

The outputs differ also strongly between these health care institutions. In the CSPS Yevedougou only 40 children were born within seven months, whereas women gave birth to 897 babies in Barani CSPS. Most outpatient visits (4,654) were reported in Djibasso, only 976 in Bagala. Among the output variables the number of vaccinations shows the widest variability. Excluding the new facilities there were 41,231 cases in Barani and 2,028 in Kononkoira.

The analysis of the Nouna Health District Household Survey showed that 4,495 people (61.2%) are Muslims, 1,997 (27.2%) were Catholics, 337 (4.9%) Protestants and 480 (6.5%) were Animists. The most important ethnic group are the Dafing with 2,867 (39%) members. Other included ethnics were the Bwaba (1,740 people, 23.7%), the Mossi (1,227 people, 16.7%). the Samo (742 people, 10.1%) and the Peulh (680 people, 9.2%).

Based on the data on inputs and outputs we could calculate the first stage of the DEA model, the results of which are presented in the next sub-section.

### First stage DEA

Results obtained by the application of the output-orientated DEA approach according to constant returns to scale (CCR) are presented by table 2.

**Table 2:** Efficiency scores and reference and reference sets according CCR assumption, 3 inputs and 3 outputs (left), and 4 inputs and 4 outputs (right) respectively

3 inputs & 3 outputs				4 inputs & 4 outputs			
**DMU**	**Score**	**Reference set**	**λ**	**DMU**	**Score**	**Reference set**	**λ**

Bagala	0.5115	Dara	0.0001	Bagala	0.5207	Dara	0.1089
		Berma	0.0276			Bomborokuy	0.0001
		Doumbala	0.0001			Nian	0.0001
						Dembo	0.0001
						Berma	0.1989

Barani	1			Barani	1		

Berma	1			Berma	1		

Bomborokuy	1			Bomborokuy	1		

Borekuy	0.7310	Berma	0.3304	Borekuy	0.7703	Dembo	0.1104
		Doumbala	0.2575			Berma	0.3070
						Bourasso	0.0001

Bourasso	1			Bourasso	1		

Dara	1			Dara	1		

Dembo	1			Dembo	1		

Djibasso	0.5834	Berma	0.8827	Djibasso	0.5867	Nian	0.0001
		Barani	0.1390			Berma	0.8071
		Bourasso	0.3897			Barani	0.1369
						Bourasso	0.3834

Dokuy	0.5750	Barani	0.2938	Dokuy	0.5844	Dembo	0.0001
		Bourasso	0.1532			Barani	0.21551
						Bourasso	0.2029

Doumbala	1			Doumbala	1		

Gassingo	0.3693	Bourasso	0.1931	Gassingo	0.7624	Nian	0.0001
		Ira	0.0001			Bourasso	0.1826

Goni	0.4444	Berma	0.3535	Goni	0.9934	Nian	0.0001
						Bourasso	0.2295
						Nouna	0.0001

Ira	1			Ira	1		

Kienekuy	0.8358	Berma	0.2824	Kienekuy	0.8429	Dembo	0.2042
		Doumbala	0.5063			Berma	0.4364
						Barani	0.0001
						Doumbala	0.0001

Konankoira	0.4018	Berma	0.4788	Konankoira	0.8018	Dara Bourasso	0.4625 0.1647
						Nouna	0.0394

Konkuy Koro	0.6578	Barani	0.2611	Konkuy Koro	0.7437	Dembo	0.2322
		Bourasso	0.2284			Berma	0.0001
						Barani	0.0001
						Bourasso	0.1865

Koro	0.8362	Dembo	0.5192	Koro	0.611	Dara	0.3958
		Barani	0.3573			Nian	0.0001
						Dembo	0.5996

Lekuy	1			Lekuy	1		

Nian	0.9047	Berma	0.8076	Nian	1		
		Barani	0.0001				
		Bourasso	0.0876				

Nouna	0.6567	Dara	0.1309	Nouna	1		
		Berma	0.3366				
		Doumbala	0.3082				

Sono	0.5764	Dembo	0.1255	Sono	0.6457	Dembo	0.2563
		Berma	0.0001			Berma	0.1310
		Barani	0.2546			Barani	0.0001
						Bourasso	0.0001

Toni	0.7911	Bourasso	0.5808	Toni	0.9069	Dara	0.0001
		Ira	0.2482			Nian	0.0001
						Berma	0.1567
						Nourasso	0.5537
						Nouna	0.0001

Werebere	0.8493	Berma	0.3298	Werebere	0.8611	Dembo	0.1675
		Doumbala	0.3773			Berma	0.4236
						Bourasso	0.0001

Yevedougou	0.2454	Berma	0.2734	Yevedougou	0.5996	Nian	0.0001
						Bourasso	0.2090
						Nouna	0.0001

The primary care facilities are arranged alphabetically. Results both from including 4 inputs and 4 outputs (right part of the table) and excluding vaccination (left) are shown. In general, the scores, which are recorded in columns two and six, are quite similar. DMUs which are efficient in the reduced model are also best practice in the comprehensive one. However, in the latter model the facilities of Nian and Nouna are additionally relative efficient. Eleven of the twenty-five DMUs (44%) are best practice (score = 1) in the extended approach, and nine (36%) in the reduced one, respectively. It can be seen that Gassingo is the worst performer excluding vaccination (0.3693), whereas Bagala shows the largest inefficiencies in the comprehensive one with a score value of 0.5207. The columns "reference set" and "λ" include the corresponding reference units for the inefficient DMUs and the λ values, which are the raw weights assigned to peer units when solving the DEA approach. The higher the contribution, the closer in performance is the peer to the unit under consideration. As shown in the table, e.g. Bagala can virtually become efficient by combining the CSPS in Dara, Bomborokuy, Nian, Dembo and Berma as peers with raw weights of λ = 0.1089, λ = 0.0001, λ = 0.0001, λ = 0.0001 and λ = 0.1989.

In the follow-up analyses we concentrated on the extended model, because neglecting immunisation would be mean omitting the most expensive input of CSPS services. Based on the assumption of variable returns to scale (BCC), 17 of 25 primary care facilities (68%) are relatively efficient. This reflects that the envelopments' surface differs depending on the scale assumptions that underpin the model. Table 3 presents the efficiency results for the remaining eight inefficient CSPS under this most flexible returns to scale assumption.

**Table 3:** Efficiency scores, input-output original data and projection for inefficient CSPS according to the VRS assumption

DMU	Score	Input/Output	Original data	Projection	Difference (%)
Dokuy	0.8690	Personnel costs	5,208	4,526	-13.10
		Area	114	99	-13.10
		Equipment	5,278	1,709	-67.61
		Vaccine	16,214	14,089	-13.10
		Outpatient	1,247	1,727	38.46
		Deliveries	237	391	65.15
		Other	2,485	2,485	0.00
		Vaccination	8,401	8,401	0.00

Kienekuy	0.9733	Personnel costs	3,566	3,471	-2.67
		Area	189	153	-19,21
		Equipment	1,921	1,870	-2.67
		Vaccine	14,635	13,449	-8.10
		Outpatient	1,904	1,9949	2.36
		Deliveries	413	413	0.00
		Other	2,725	2,725	0.00
		Vaccination	4,987	4,987	0.00

Konankoira	0.8126	Personnel costs	4,680	3,685	-21.27
		Area	158	128	-18.74
		Equipment	2,019	1,211	-40.00
		Vaccine	7,861	6,388	-18.74
		Outpatient	1,445	1,445	0.00
		Deliveries	249	249	0.00
		Other	1,172	2,541	116.84
		Vaccination	2,028	3,730	83.95

Konkuy Koro	0.9162	Personnel costs	8,074	5,205	-35.54
		Area	102	93	-8.38
		Equipment	2,635	2,414	-8.38
		Vaccine	9,134	8,368	-8.38
		Outpatient	1,511	2,220	46.90
		Deliveries	214	227	5.88
		Other	2,568	2,568	0.00
		Vaccination	2,648	3,348	26.44

Koro	0.9630	Personnel costs	6,264	5,301	-15.37
		Area	200	186	-6.77
		Equipment	3979	2,030	-48.97
		Vaccine	13.450	12,952	-3.70
		Outpatient	1,612	2350	45.76
		Deliveries	391	479	22.61
		Other	5,600	5,600	0.00
		Vaccination	7,817	7,817	0.00

Sono	0.7966	Personnel costs	4,853	3865,84	-20.34%
		Area	142	113	-20.34%
		Equipment	4,628	1,222	-79.59%
		Vaccine	15.100	12,029	-20.34
		Outpatient	1,452	1,525	4.99
		Deliveries	322	343	6.37
		Other	2,713	2,713	0.00
		Vaccination	3,943	4,334	9.90

Toni	0.9403	Personnel costs	6,137	5,592	-8.88
		Area	105	99	-5.97
		Equipment	3,086	2,768	-10.29
		Vaccine	10.487	0,862	-5.97
		Outpatient	2,972	2,972	0.00
		Deliveries	277	277	0.00
		Other	2,169	2912	34.27
		Vaccination	4,320	4,320	0.00

Yevedougou	0.8828	Personnel costs	4,375	3,124	-28.59
		Area	271	112	-58.67
		Equipment	3255	1,066	-67.26
		Vaccine	3,182	2,809	-11.72
		Outpatient	825	1,067	29.33
		Deliveries	40	74	85.00
		Other	544	761	39.89
		Vaccination	588	1,206	105.10

Therefore, the resulting efficiency scores which are reported in the second column are much higher than the corresponding values in table 2. The third column lists the included input and output variables, with the original data aside. The projection column presents the results of the DEA calculations. A DMU is BCC efficient if it has no input excesses and no output shortfalls. If so, the difference between original data and projection is 0.00%. The inefficient CSPS should improve their productivity and make better use of their resources. The improvements needed to reach it are listed in the last column.

The DEA projections suggest that the inefficient units were too large for being efficient. The results indicate that e.g. the personnel costs (area) in Dokuy CSPS are 13.10% (16.28%) too high for being efficient. Similarly, efficiency can theoretically be attained if the output values of outpatient visits and deliveries are increased by 38.46% and 68.15%, respectively. Because the DEA calculations based on the BCC model incorporate the effects of DMU size on efficiency, a primary care facility only needs to be purely technically efficient to be considered as efficient. Thus, if the ratio efficiency-CCR/efficiency-BCC is calculated the scale efficiency can be determined. The corresponding scores are presented in Table 4.

**Table 4:** Scale efficiency scores

CSPS	Scale efficiency
Bagala	0.5207

Barani	1

Berma	1

Bomborokuy	1

Borekuy	0.7703

Bourasso	1

Dara	1

Dembo	1

Djibasso	0.5867

Dokuy	0.6726

Doumbala	1

Gassingo	0.7624

Goni	0.9934

Ira	1

Kienekuy	0.8660

Konankoira	0.9868

Konkuy Koro	0.8118

Koro	0.9980

Lekuy	1

Nian	1

Nouna	1

Sono	0.7715

Toni	0.9645

Werebere	0.8611

Yevedougou	0.6793

The corresponding implication for e.g. the health facility in Bagala is that it operates locally efficient (BCC efficient, pure technical efficiency = 1), whereas its overall technical inefficiency is caused by its failure to achieve scale efficiency, represented by SE = 0.5207. In particular, 10 of the 13 scale inefficient CSPS had their technical efficiency scores higher than the scale efficiency scores. This implies that the overall inefficiency is primarily due to the scale inefficiency, i.e., they are either too big or the demand for services is too low. The latter calls for a thorough analysis of demand-side factors influencing the efficiency of the primary care units. The results will be presented in the next sub-section.

#### Second stage DEA

Table 5 presents an overview of the environmental variables which were used in the second stage. To control for the influence of religion and ethnic groups, respectively, the corresponding categories were implemented as dummy variables. In table 6 descriptive statistics of the environmental variables are provided.

**Table 5:** Overview of environmental variables

Distance	distance in km to the next health centre
HHmemb	Number of household members
**Livestock**	
AnInd	Value of animal ownership at personal level (in F CFA)
AnHH	Value of animal ownership at household level (in F CFA)
AnHHead	Value of animal ownership at household level per capita (in F CFA)
**Durable household goods**	
GoodInd	Value of durable household goods at personal level (in F CFA)
GoodHH	Value of durable household goods at household level (in F CFA)
GoodHHead	Value of durable household goods at household level per capita (in FCFA
**Ethnic Groups**	
EBwa	1, if Bwaba
EDaf	1, if Dafing
EMos	1, if Mossi
EPlh	1, if Peulh
ESam	1, if Samo
**Religion**	
RAni	1, if Animist
RCat	1, if Catholic
RMus	1, if Moslem (incl. Ouhabien)
RPrt	1, if Protestant

**Table 6:** Descriptive statistics of environmental variables

Variable	Mean	Standard Deviation	Minimum	Maximum
Distance	12.38	12.76	0	62

HHmemb	12.31	8.83	1	57

AnInd	47,230	383,208	0	23,136,300

AnHH	633,827	1,499,547	0	23,663,300

AnHHead	47,574	130,228	1	2,746,000

GoodInd	90,634	1,321,839	0	64,485,000

GoodHH	1,175,234	4,717,385	0	65,435,000

GoodHHead	92,530	484,649	1	13,900,000

EBwa	0.237	0.425	0	1

EDaf	0.390	0.488	0	1

EMos	0.167	0,373	0	1

EPlh	0.092	0.290	0	1

ESam	0.101	0.301	0	1

RAni	0.065	0.247	0	1

RCat	0.272	0.445	0	1

RMus	0.612	0.487	0	1

RPrt	0.049	0.215	0	1

Note: Number of observations: 7,346.

The financial variables are expressed in F CFA, the regional currency. According to our data the livestock value of the richest individual was 23,136,300 F CFA (43,334 US$), on average at the household level per capita was 47,574 F CFA (88 US$). The highest value of individual owned durable household goods was 64,485,000 F CFA (119,942 US$). Taking into account that households in the sample have a size up to 57 members, with mean of about 12 members, the average value of household goods per capita at that level was 92,530 F CFA (177 US$). Keeping in mind that the used market price data do not account for depreciation and illness, the data can be interpreted as reflection of the extreme poverty in that district.

Correlation coefficients between all variables included in the analysis were calculated. Generally speaking, there is no or only weak pair wise correlation between the environmental variables. Additionally, multicollinearity of the used independent variables was checked by calculating the variance inflation factor for each variable. Excluding one category of religious belief and ethnic group helps to avoid this problem and results in tolerance levels above 0.2.

In Table 7 and 8 we report the findings of three different estimates of the 2^nd ^stage analysis. We chose the normal censored Tobit and the truncated regression model as starting point.

**Table 7:** Second stage estimation results from censored and truncated regression

	Censored		Truncated	
	
	Coefficient	*p *value	Coefficient	*p *value r
Distance	0.107***	(0.001)	0.114***	(0.001)
AnInd	0.001	(0.969)	0.001	(0.763)
AnHH	-0.001***	(0.000)	-0.001*	(0.008)
AnHHead	-0.017*	(0.000)	-0.013**	(0.016)
GoodInd	0.001	(0.510)	-0.003	(0.469)
GoodHH	0.032**	(0.000)	0.096**	(0.001)
GoodHHead	-0.001*	(0.000)	-0.001**	(0.001)
EBwa	-0.014	(0.313)	-0.064**	(0.004)
EDaf	-0.047***	(0.000)	-0.115***	(0.000)
EPlh	-0.038*	(0.016)	-0.251***	(0.002)
ESam	0.192***	(0.000)	0.083***	(0.000)
Rani	0.076**	(0.001)	0.174***	(0.000)
Rcat	-0.107***	(0.000)	-0.169***	(0.000)
Rmus	0.209***	(0.000)	0.218***	(0.000)
Constant	0.180***	(0.000)	0.373***	(0.038)

Sigma	0.296***	(0.003)	0.273***	(0.000)

Log Likelihood	-2823.7959		1564.8673	
Wald	122.43	(0.000)	869.53	(0.000)

Note: The table presents estimates using a Tobit and three specifications of the bootstrap censored based methodology. The dependent variable is DEA score based on the CCR technology with four input and four output variables. The superscripts: *, **, and *** denote significance at the 10%, 5% and 1% levels, respectively.

**Table 8:** Second stage estimation results from bootstrap truncated regression

	Model I			Model II			Model III		
	
		95% Confidence interval			95% Confidence interval			95% Confidence interval	
	Coefficient	Lower	Upper	Coefficient	Lower	Upper	Coefficient	Lower	Upper
Distance	0.135***	0.117	0154	0.137***	0.119	0.155	0.146***	0.126	0.166
AnInd	3.52e -08	-2,50e-08	9.55e-08	3.53e-0.8	-2.54-08	9.59-08	-4.89e-08	-1.11e-07	1.33e-08
AnHH	-7.95e-08	-9.42e-08	-6.47e-08	-7.93e-08	-9.42e08	-6.45e	-	-	-
AnHHead	-0.006**	-0.001	-0.000	-0.001*	-0.001	-0.000	-0.001**	-0.001	-0.0000
GoodInd	-0.001	-2.76e-08	2.33e-08	-0.000	-2.84e-08	2.41-08	1.08e-08	-1.82e-08	3.97e-08
GoodHH	0.125**	0.097	0.153	0.000**	9.74e-09	1.54e-08	-	-	-
GoodHHead	-0.005***	-0.005	-0.004	-0.005***	-0.005	-0.004	-0.0005***	-0.001	-0.004
EBwa	-0.064*	-0.115	-0.014	-0.041	-0.006	-0.870	-0.530	-0.107	0.001
EDaf	-0.115***	-0.144	-0.086	-	-	-	-0.105***	-0.136	-0.742
Emos	-	-	-	0.104***	0.731	0.134	-	-	-
EPlh	-0.251***	-0.310	-0.193	-0.145***	-0.202	-0.870	-0.350***	-0.415	-0.285
ESam	0.083***	0.055	0.110	0.186***	0.164	0.216	0.105***	0.077	0.133
RAni	0.174***	0.239	0.324	-0.345	-0.457	-0.233	0.182	0.024	0.34
RCat	-0.169	-0.271	-0.674	-	-	-	-0.201	-0.311	-0.091
RMus	0.218***	0.122	0.313	0.472**	0.0146	0.798	0.257***	0.151	0.363
RPrt	-	-	-	0.166**	0.271	0.606	-	-	-
Constant	0.372***	0.274	0.471	0.439	0.397	0.481	0.320	0.208	0.431

Sigma	0.276***	0.264	0.288	0.277***	0.264	0.289	0.288***	0.275	0.302

Note: The table presents estimates based on normal censored and truncated models. The dependent variable is DEA score based on the CCR technology with four input and four output variables. Inference is based on confidence intervals obtained from 2000 bootstrap iterations. The superscripts: *, **, and *** denote significance at the 10%, 5% and 1% levels, respectively; standard errors in parenthes

The Tobit regression model predicting DEA scores by the spatial and socio-economic variables is statistically significant (χ^2 ^= 1630.93, df = 14). Many of the independent variables in the model are also statistically significant at the 0.01 level. Since the efficiency measure ranges between 0 and 1, we inverted the dependent variable and subtracted one. Thus the sign of the coefficients are reversed - a positive coefficient implies an inefficiency increase whereas a negative coefficient means an association with inefficiency decline, thus increased efficiency. Longer distances to rural primary care facilities seems to have a clear negative impact on the efficiency, which is reflected in the positive sign of the corresponding coefficient.

Also the affiliation with a special religious belief can be linked with the performance of health facilities, because of the corresponding actual demand of modern health care or not. However, caution is required concerning the interpretation of the coefficients' signs: In the section "religion" the presented values have to be interpreted with reference to Protestants, in the category "ethnic group" the corresponding benchmark are the Mossi. Keeping that in mind, the criterion "Animist" is linked with an inefficiency increase compared to the Protestants. Most of the ethnic groups (Bwaba, Dafing and Peulh) in Nouna Health District seem to contribute to an efficiency increase in comparison to the Mossi, whereas the result for the Bwaba is not significant at all.

The findings concerning the livestock and the household goods variables are less clear. Neither personal ownership of animals nor of durable goods as a proxy of income and wealth (ability to pay) seem to have an influence on the efficiency of a CSPS. Unlike that, the household level is much more important. However, the result for the wealth of a household per se without considering the number of family members is significant on a higher level. The squared correlation between the observed and predicted efficiency score (McKelvey & Zavainona 's pseudo R^2 ^) is 0.219, indicating that the included predictors account for nearly 22% of the variability in the outcome variable; which is rather weak. The value of the ancillary statistic "sigma" (0.296) can be compared with the standard deviation of the independent variable which is 0.254. This reflects an increase.

The results from a truncated regression model based on the same specification are presented in the right part of the table. In principle, most of the findings are similar. The signs are the same but the significance of the estimated coefficients has risen. Regarding to the significance of livestock and the ownership of durable goods variables it can be seen that under this estimation technique the importance of per capita specifications has gained more importance. For example, a unit change in the value of household livestock per capita leads to a 1.3% increase in the predicted score level. The sigma value, which can be used for assessing the fit, suggests a very modest reduction in comparison to the Tobit model.

Table 8 reports the estimation results from the bootstrap procedures linked with a truncated regression as described in the method's section.

We used three different model specifications. The variables which are used as independent variables in model I are identical to the non-bootstrapped regressions. By contrast, the reference value of the ethnic groups and religious confession is changed in model II. Estimation model III differs from model I in terms of cancelling the aggregated household variables. It is obvious, that the estimated coefficients are very similar irrespective of the applied specification. Moreover, they are close to the estimates derived from the more usual Tobit procedure, and, very importantly, they are highly significant. Analogously, the fit of all of them is nearly the same, whereas model I provides the best fit, as can be seen by the lowest value of sigma.

## Discussion

The efficient use of scarce resources is especially important in poor countries. Data Envelopment Analysis is an appropriate tool to compare the performance of similar units, e.g. health facilities at the same level of care.

The first-stage findings identify clearly some inefficient primary care facilities and their corresponding peers. Generally speaking, the results are in line with the existing studies on the performance of health facilities. Most of the published DEA studies report a quite high mean efficiency score. For example Hollingsworth [31] reported in a recent review on the results of efficiency measurement worldwide across different types of hospitals a mean efficiency score of 0.835 and the corresponding median of 0.85. The average score of the primary care facilities in Nouna Health District based on constant returns to scale is 0.862. However, this result is higher than the corresponding findings from similar facilities in Zambia (0.619) reported by Masiye et at. [50], and in Sierra Leone (0.78) studied by Renner et al. [51] respectively.

It is often supposed, that facilities in Africa are not very efficient. There is no contradiction between this statement and our findings, as the first one is based on the concept of absolute efficiency, but DEA is linked with relative efficiency. Thus a high DEA score does not mean that these facilities are 'well managed' and that there would be little scope for improvement. Most of the DEA studies conducted in sub-Saharan Africa report a very high share of inefficient facilities. For example Akzahli et al. [21] found out that 78% of the analysed primary care facilities in Ghana were technically inefficient and Kirigia et al. 2001 [52] report that 70% of the studied primary health care clinics in South Africa were inefficient. If the studied facilities are quite homogenous their performance might be similar. This is the case in our study: there is obviously some deviation, but in general the primary care facilities are quite similar - especially in terms of underutilization and excess capacities [53]. Thus only 56% are below the best-practice frontier, which is identical to the result of Kririgia et al. [23].

Our findings of the average scale efficiency score (0.8898), which is nearly the same as in the case of 89 randomly sampled primary care facilities in Ghana (86%) analysed by Akzali et al. [54], allows for an interesting remark: The average size of the CSPS in Nouna Health District is not far from relative efficiency, although an additional 11% productivity gain would be feasible - assuming no other constraining factors - provided they adjusted their services to an optimal scale.

Second stage DEA is the logical extension of traditional performance measurement by considering non-discretionary environmental parameters which might influence the facilities' performance. So far, only one study tried to take these factors into account in the case of sub-Saharan Africa. Akzali et al. [21] estimated the impact of several economic and structural factors on the DEA efficiency measure of 88 randomly selected health facilities. Based on a logistic regression they showed that variables like "access to safe drinking water" or "staff incentives" matter. However, these factors are rather general and do not explicitly focus at barriers for health care seeking. They are consistent with the applied input-oriented approach. In the case of Nouna Health District we combined the output-oriented approach at the first stage with factors influencing the demand side at the second stage.

The high significance of the included spatial, economic and cultural variables induces some clear messages, which can be directly linked with the existing literature. From an ethical point of view it is not appropriate to try to reduce waste of resources by downsizing or closing of some of the CSPS with a worse performance [55], which is suggested by some other DEA-papers which focus at sub-Saharan Africa health facilities e.g. [21,19], but to take better advantage of the existing capacities. Apparently it will not be possible that most of the Millennium Development Goals will be met by 2015 in Africa. From a medical point of view it is well-known that there is a large latent demand for health care services in Africa; but the actual demand for modern health care is low. The corresponding question is how to increase utilisation rates. Flessa [6] showed that three steps have to be accomplished depending each on various factors. First, a need has to be perceived in the population. In the case of an illness, the condition has to be regarded as abnormal and modifiable. This depends, among others, on the health status of the patient's surrounding. A need will become a want, if means are defined, which are considered capable to satisfy the need. This depends, however, on the concept the patient has about the disease and their understanding of an appropriate treatment by western or traditional health care. Finally, the turning of a want into a demand depends on the affordability, the accessibility, the quality and on the personal priority setting. Our findings confirm this concept. Geographical accessibility, which is approximated by the distance variable, is of great importance for the decision to visit a CSPS and thus for the number of patients. Thus it is the health policy's task to improve the spatial allocation of primary care facilities in order to induce additional demand. This has been done in Nouna Health District during the last years, but there are still some improvements possible [56].

The results of this study are also in line with analyses which focus at the importance of wealth, to the corresponding ability to pay and the consequences for health-care seeking, e.g. [57]. According to our estimates the importance of total household resources is more significant than total household assets per capita, a result which could be questioned.

Our estimations also show that cultural attributes and the religious confession of the population within the catchment area of a CSPS matter. Marschall and Flessa [24] pointed out that in 2004 the primary care facility Ira performed badly because the treatment was not in line with their conservative Islamic belief. After some changes in the facilities' staff, the CSPS was re-accepted by this part of the population. Our results show, that the variable "Moslem" can be associated with a negative influence on efficiency. Unfortunately it is not possible on the basis of our database to distinguish between different subgroups. The same argument seems to be valid for some ethnic groups. Their traditional concepts have to be linked with the provision of modern health care.

Most of the costs at the level of primary care in the Nouna Health District are fixed and there is much idle capacity. Thus increased utilization of these services is possible without much additional costs [58]. Consequently, both in this district and in health facilities with a similar background, global targets like the MDGs might be attainted with little additional money [59].

There are several methodological suggestions for conducting 2^nd ^stage analysis [43]. To meet elementary statistical concerns, bootstrapping has become more important. Whereas in the aftermath of the work by Simar and Wilson [38] bootstrapping is increasingly used both at the first and at the second stage, e.g. [60] and [61], we only used it on the second one, because our database at the first level included information from all facilities and not just a small sample population. Our corresponding results relating to econometric methods suggest that either of the applied (non) bootstrapped applications can be justified as the appropriate method for evaluating the impact on efficiency. The estimated Tobit and truncated coefficients are within or near the bootstrapped confidence intervals. Furthermore, the results are quite robust across different specifications. This kind of observation is not unique. According to Alfonso and St. Aubyn [60] this can be interpreted as additional support for the obtained results.

## Conclusions

This paper investigates the performance of all primary care facilities in a rural health district in Burkina Faso and the linkages with spatial and socio-economic variables on the demand side by using DEA and regression analyses. In the African culture it would be almost impossible to close down health care institutions, and in Nouna health district this would result in inacceptable travel distances for patients. Consequently, the health policy decision-makers should aim at increasing the efficiency of these facilities by providing incentives for the people in the catchment area for increasing modern healthcare seeking. One important first step is to overcome barriers that prevent people from turning wants into actual demand. It is not enough that within the framework of the MDG campaign new primary care facilities are built, the people should also be attracted by a convincing strategy. In this regard we demonstrated that the discussion about overcoming demand side barriers and improving efficiency are just the two sides of the same coin.

This study has some limitations: First, the study area of the 1^st ^stage and the 2^nd ^stage analysis differ. Because the Nouna Health District Household Survey does not cover Kossi totally, it was only possible to explain the calculated DEA efficiency scores of eight primary care facilities by corresponding demand-side data. Second, there are further demand-side factors which might have a significant impact for health-care seeking [62], e.g. priority or urgency of need. Unfortunately it was not possible to link the disposable data with specific CSPS. We also did not consider quality aspects of health care provision. Previous investigation in the study area showed that people did not accept some health care services because of the perceived low quality of care [63] and resort to traditional medicine or self-treatment [64]. This was also pointed out by [65]. It was also not possible to fit the quality-related variables which are included in the household survey in our study. Furthermore education matters for the acceptance of modern health care. We did not include corresponding variables into our regression analysis because the formal education in Nouna Health District is on a very low level and according our estimation strategy it was not useful to inflate the estimation with an additional bundle of dummy variables. In addition, there is a high correlation with the livestock and the household goods variables.

The obvious next step should be a thorough analysis of the DEA score dynamics in Nouna Health District. By using more comprehensive provider and household data based on a longer period of time it is possible to investigate the impact of health policy changes on the CSPS efficiency.

## Competing interests

The authors declare that they have no competing interests.

## Authors' contributions

The authors did the research jointly.
